# Untargeted Lipidomic Profiling Reveals Lysophosphatidylcholine and Ceramide as Atherosclerotic Risk Factors in *apolipoprotein E* Knockout Mice

**DOI:** 10.3390/ijms24086956

**Published:** 2023-04-09

**Authors:** Shi-Hui Law, Hua-Chen Chan, Guan-Ming Ke, Swetha Kamatam, Gopal Kedihithlu Marathe, Vinoth Kumar Ponnusamy, Liang-Yin Ke

**Affiliations:** 1 Department of Medical Laboratory Science and Biotechnology, College of Health Sciences, Kaohsiung Medical University, Kaohsiung 80708, Taiwan; 2 Department of Medical Laboratory Science, College of Medicine, I-Shou University, Kaohsiung 84001, Taiwan; 3 Center for Lipid Biosciences, Department of Medical Research, Kaohsiung Medical University Hospital, Kaohsiung 80708, Taiwan; 4 Graduate Institute of Animal Vaccine Technology, College of Veterinary Medicine, National Pingtung University of Science and Technology, Pingtung 91201, Taiwan; 5 Department of Studies in Biochemistry and Molecular Biology, University of Mysore, Manasagangothri, Mysuru 570006, India; 6 Department of Medicinal and Applied Chemistry, Research Center for Precision Environmental Medicine, Kaohsiung Medical University, Kaohsiung 80708, Taiwan; 7 Graduate Institute of Medicine, College of Medicine, Kaohsiung Medical University, Kaohsiung 80708, Taiwan

**Keywords:** lysophosphatidylcholine (LPC), ceramide (CER), cholesteryl ester (CE), triglyceride (TG), lipidomic, mass spectrometry, *apolipoprotein E* (*apoE*) knockout, atherosclerotic cardiovascular disease (ASCVD), atherosclerosis

## Abstract

Despite the availability and use of numerous cholesterol-lowering drugs, atherosclerotic cardiovascular disease (ASCVD) remains the leading cause of mortality globally. Many researchers have focused their effort on identifying modified lipoproteins. However, lipid moieties such as lysophosphatidylcholine (LPC) and ceramide (CER) contribute to atherogenic events. LPC and CER both cause endothelial mitochondrial dysfunction, leading to fatty acid and triglyceride (TG) accumulation. In addition, they cause immune cells to differentiate into proinflammatory phenotypes. To uncover alternative therapeutic approaches other than cholesterol- and TG-lowering medications, we conducted untargeted lipidomic investigations to assess the alteration of lipid profiles in *apolipoprotein E* knockout (*apoE*^−/−^) mouse model, with or without feeding a high-fat diet (HFD). Results indicated that, in addition to hypercholesterolemia and hyperlipidemia, LPC levels were two to four times higher in *apoE*^−/−^ mice compared to wild-type mice in C57BL/6 background, regardless of whether they were 8 or 16 weeks old. Sphingomyelin (SM) and CER were elevated three- to five-fold in *apoE*^−/−^ mice both at the basal level and after 16 weeks when compared to wild-type mice. After HFD treatment, the difference in CER levels elevated more than ten-fold. Considering the atherogenic properties of LPC and CER, they may also contribute to the early onset of atherosclerosis in *apoE*^−/−^ mice. In summary, the HFD-fed *apoE*^−/−^ mouse shows elevated LPC and CER contents and is a suitable model for developing LPC- and CER-lowering therapies.

## 1. Introduction

Cardiovascular diseases (CVDs) remain the leading cause of mortality worldwide. In 2019 alone, an estimated 17.9 million individuals died from CVD, accounting for 32% of all global deaths [1,2]. The current strategies for CVD prevention primarily focus on lowering plasma levels of low-density lipoprotein-cholesterol (LDL-C) by statins [3,4]. Although intensive statin therapy has been shown to lower LDL-C levels and reduce the incidence of secondary CVD, it also boosts the risk of adverse effects and drug intolerance [5,6]. *Herba patriniae*, a phytomedicine, has been identified as a potential anti-atherosclerosis candidate by modulating the glycerophospholipid metabolic pathway [7]. In recent years, Proprotein Convertase Subtilisin Kexin Type 9 (PCSK9) inhibitors have emerged to be an alternative approach for the prevention of CVDs [8]. PCSK9, a protease that removes LDL receptors (LDLR) and its inhibition by alirocumab, evolocumab, and inclisiran aggressively increase LDL receptor levels for LDL-C clearance in the liver [9,10]. However, an extremely low LDL-C level is associated with an increased risk for all-cause death [11,12,13]. Thus, CVD remains a challenging medical issue.

For more than three decades, researchers have focused their attention on identifying modified lipoproteins including oxidized LDL (oxLDL) and electronegative LDL (LDL(−)), followed by their signaling events. Signaling through the lectin-like oxidized LDL receptor-1 (LOX-1) [14,15], oxLDL is an essential component in the pathogenesis of atherosclerosis and CVD [16,17,18]. Synthetic LOX-1 inhibitors and neutralizing antibodies have shown encouraging benefits in animals, warranting further research in this field [19]. In contrast to in vitro preparations, LDL(−) is isolated from human plasma using fast-protein liquid chromatography and shown to be mildly oxidized [20]. Endogenously generated LDL(−) is positively associated with the severity of CVD [21,22,23,24]. Lysophosphatidylcholine (LPC), an abundant component in both oxLDL and LDL(−), is elevated in familial combined hyperlipidemia and cardiovascular diseases [25,26]. In addition, ceramide and triglyceride (TG) are highly elevated in LDL(−) and play crucial roles in atherosclerosis [27,28].

To evaluate the efficacy of new therapeutic approaches, notably targeting the aforementioned lipid species, having a suitable animal model is of utmost importance. *Apolipoprotein E*-deficient (*apoE*^−/−^) mice are prone to develop atherosclerotic plaques and are thus commonly employed in CVD research [29]. NADPH oxidase activator 1 (NOXA1), NADPH oxidase 2 (Nox2), and reactive oxygen species (ROS) are overproduced in different cellular compartments of *apoE*^−/−^ mice. This leads to mitochondrial damage, endothelial dysfunction, and atherosclerosis. In contrast, the inhibition of ROS decreases atherosclerotic lesion formation [30,31]. A high-fat diet (HFD) may expedite disease progression. HFD-fed *apoE*^−/−^ mice show insulin resistance and exhibit steatohepatitis after 8 weeks of HFD consumption [32]. Furthermore, HFD-fed *apoE*^−/−^ male mice develop hypercholesterolemia, with a four-fold higher level of TG in plasma. However, the exact breakdown of the LDL-lipid components in the plasma of HFD-fed *apoE*^−/−^ male mice remains unclear. In this regard, we have analyzed other lipid components, including LPC and CER in both *apoE*^−/−^ and HFD-fed *apoE*^−/−^ mice.

In recent years, the mass spectrometry (MS)-based lipidomic assay has emerged as a promising approach for better understanding of CVD pathophysiology and exploring etiological determinants [33,34]. Sophisticated bioinformatics and biostatistics could aid in the discovery of new lipid mediators, metabolites, or disease biomarkers [35,36]. Different lipid species can be distinguished using ACQUITY^®^ ultra-performance liquid chromatography (UPLC) equipped with a charged surface hybrid (CSH) C18 column. The separation is based on the length of the acyl chain, number, position, and geometry of double bonds [28]. UPLC-MS data can be processed using Progenesis QI^®^ software for lipid identification and quantification [37]. We sought to explore untargeted lipidomic analysis for *apoE*^−/−^ and HFD-fed *apoE*^−/−^ mice in contrast to wild-type controls employing this modern approach. By analyzing total lipid extracts of plasma and LDL, we aimed to detect molecular fingerprints of *apoE*^−/−^ and HFD-fed *apoE*^−/−^ mice for atherosclerosis and CVD.

## 2. Results

### 2.1. Biochemical Markers Relevant to Atherosclerosis in Three Groups of Mice at Basal Level (at 8th Week)

Table 1 summarizes the basal values (8 weeks old) for all the parameters studied in all three groups of mice, although at this stage the diet regime had not started yet. Body weights were significantly increased in both groups (Group II and Group III) of *apoE*^−/−^ mice when compared to wild-type control mice (*p* < 0.05, Table 1). However, there were statistically significant differences between the two groups of *apoE*^−/−^ mice. In the plasma of *apoE*^−/−^ mice, the levels of glucose (*p* < 0.01), blood urea nitrogen (BUN; *p* < 0.001), total cholesterol (TC; *p* < 0.001), triglycerides (TG; *p* < 0.001), high-density lipoprotein-cholesterol (HDL-C; *p* < 0.05), and low-density lipoprotein-cholesterol (LDL-C; *p* < 0.01) were higher than in wild-type control mice (Group I). However, there was no difference in plasma biochemical parameters between the two *apoE*^−/−^ groups. The liver enzymes aspartate aminotransferase (AST, also known as glutamic oxaloacetic transaminase; GOT) and alanine aminotransferase (ALT, also known as glutamic pyruvic transaminase; GPT), the kidney function marker creatinine (Cr), and the LDL/HDL ratio of wild-type mice and *apoE*^−/−^ mice were also evaluated. However, there were no statistically significant differences between the groups (Table 1). As expected, TC levels were no different in the two *apoE*^−/−^ mice groups, which were almost three times higher than those of wild-type control mice. Although both HDL-C and LDL-C levels were increased in *apoE*^−/−^ mice, the LDL/HDL ratio did not change across groups. The two *apoE*^−/−^ mouse groups had the same TG levels, which were 1.8 times higher than those of the wild-type controls.

### 2.2. Biochemical Markers Relevant to Atherosclerosis in the Three Groups of Mice after 8 Weeks of Respective Diet (at 16th Week)

After 8 weeks of implementing the respective diets, there was no statistically significant difference in body weights between the wild-type control mice and *apoE*^−/−^ mice fed NCD. However, HFD-fed *apoE*^−/−^ mice had increased body weights (*p* < 0.001, Table 2). All the biochemical parameters studied in Table 1 were also studied after 8 weeks of feeding the respective diets (Table 2). There were statistically significant differences for all the biochemical parameters studied between Group II and Group III (Table 2). The liver enzymes (AST, ALT; *p* < 0.05, *p* < 0.001) and renal function indicators (Cr; *p* < 0.01) were all significantly increased in the HFD group when compared with the NCD group. In contrast, BUN was reduced (*p* < 0.05). The levels of lipid parameters including total cholesterol (TC; *p* < 0.001), triglycerides (TG; *p* < 0.05), HDL-C (*p* < 0.001), LDL-C (*p* < 0.001), and the ratio of LDL/HDL (*p* < 0.001) were highly elevated in the plasma of HFD-fed *apoE*^−/−^ mice. The TC levels were 1243.40 ± 48.00 mg/dL in the HFD group, about 2.5 times higher than in the NCD group (495.00 ± 29.00 mg/dL). The LDL/HDL ratio was 18.22 ± 1.07 in the HFD group, about 2.6 times higher than in the NCD group (7.06 ± 0.48).

Between Group II (*apoE*^−/−^) and Group I (wild-type) on NCD, the levels of glucose (*p* < 0.05, Table 2), total cholesterol (TC; *p* < 0.001), triglycerides (TG; *p* < 0.01), LDL-C (*p* < 0.01), the ratio of LDL/HDL (*p* < 0.01), and BUN (*p* < 0.05) were all significantly higher in the plasma of Group II when compared to wild-type mice. The TC level of Group II was about 3.2 times higher than in Group I mice. While there was no difference in HDL-C between Group II and Group I, the LDL-C concentration was significantly higher in Group II than in Group I. Thus, the LDL/HDL ratio was also significantly increased.

### 2.3. LC/MS^E^ Analysis of Plasma Lipids in All the Three Groups of Mice

**Figure 1:**
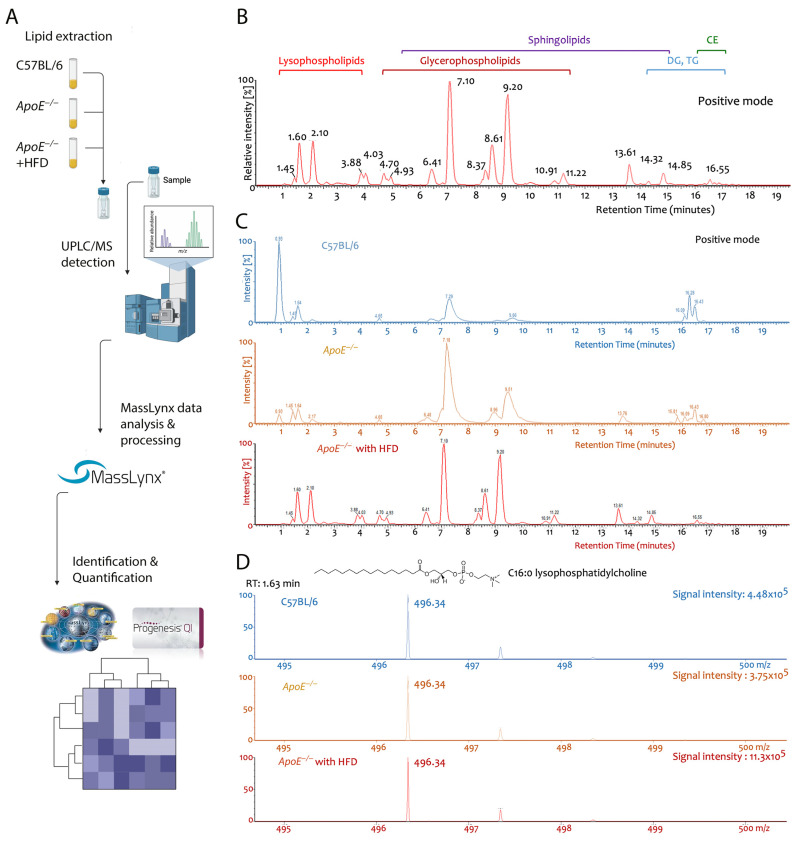
Lipidomic profiling of mouse plasma using UPLC/MS^E^. (**A**) Total lipids were extracted from the plasma from the respective groups (n = 7, each). MarkerLynx and Progenesis QI software were used to analyze and quantify the MS data. Heatmaps were used generated to display the results. (**B**) Using a CSH™ C18 column, total lipids were separated into lysophospholipids, glycerophospholipids, sphingolipids, CEs, and glycerolipids (DG, TG). The results were acquired in the positive mode. (**C**) The representative lipidomic profiling in plasma from the respective groups are demonstrated based on retention time and signal intensity. (**D**) Pattern of parent and daughter ion of LPC from the respective groups with their signal intensities. Abbreviations: *apoE*^−/−^: apolipoprotein E knockout; HFD: high-fat diet; UPLC: ultra-performance liquid chromatography; MS^E^: data-independent collection mode mass spectrometry; MS: mass spectrometry; CSH: charged surface hybrid; CE: cholesteryl ester; DG: diacylglycerol; TG: triacylglycerol.

We investigated the untargeted lipidomic profiling of mouse plasma using ultra-performance liquid chromatography/mass spectrometry (UPLC/MS^E^). MassLynx software was used to process the data, which was then quantified using MarkerLynx, Progenesis QI, and Heatmap (Figure 1A). Total lipids can be effectively categorized into lysophospholipids, glycerophospholipids, sphingolipids, sterol lipids (CE), and glycerolipids (DG, TG), according to their hydrophobicity or retention time (Figure 1B). Progenesis QI software was used to identify the primary adducts for all the lipid classes (Table A1). In addition, we investigated the overall profile of untargeted lipidomic profiling in plasma between Group I and Group II, and also between Group II and Group III mice, after 8 weeks of dietary intervention. According to our findings, the LC/MS^E^ patterns of plasma lipids were significantly altered. Lysophospholipids and glycerophospholipids tend to increase in Group III mice; however, glycerolipids seem to be decreased in HFD-fed *apoE*^−/−^ mice when compared to the wild-type and NCD-fed *apoE*^−/−^ mice (Figure 1C). We further investigated the different MS-signals according to fragments *m/z* and retention time. For example, the signal 496.34 *m/z* appeared at a retention time of 1.63 min, which corresponded to LPC(16:0). The LPC signal intensity was highly elevated in the Group III mice (11.3 × 10^5^) in comparison to Group I and Group II mice (Figure 1D).

### 2.4. Profiling Dominant Lipid Species before Diet Treatment (at 8th Week)

**Figure 2:**
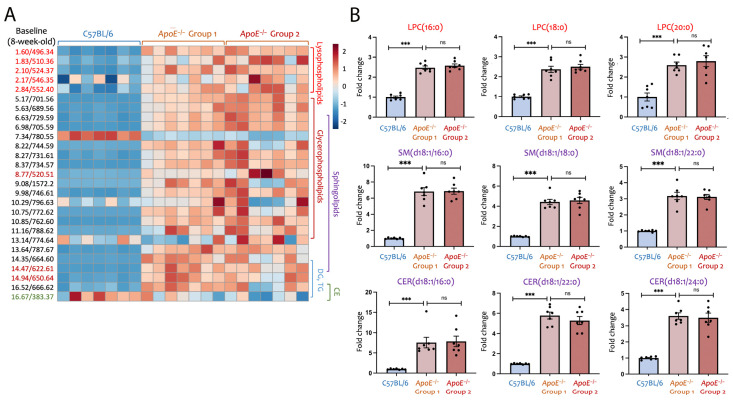
Altered lipid profile in the respective animal groups at eight weeks of age. We chose *m/z* signals with (1) higher abundance, (2) a fold change greater than 2, and (3) *p* values less than 0.001 to be the selecting markers of untargeted plasma lipidomic profiling of all the three groups of mice at the 8-week-old baseline. (**A**) A total of 27 markers were chosen to represent various lipid components between groups in a heatmap. By the exact mass and retention time, some of the 27 markers were identified. (**B**) LPC(16:0), LPC(18:0), LPC(20:0), SM(d18:1/16:0), SM(d18:1/18:0), SM(d18:1/22:0), CER(d18:1/16:0), CER(d18:1/22:0), and CER(d18:1/24:0) were compared among groups. N = 7 in each group. The data are presented in the fold change. *** *p* < 0.001, ns: not significant between groups. Abbreviations: *apoE*^−/−^: *apolipoprotein E* knockout; LPC: lysophosphatidylcholine; SM: sphingomyelin; CER: ceramide.

**Figure A1:**
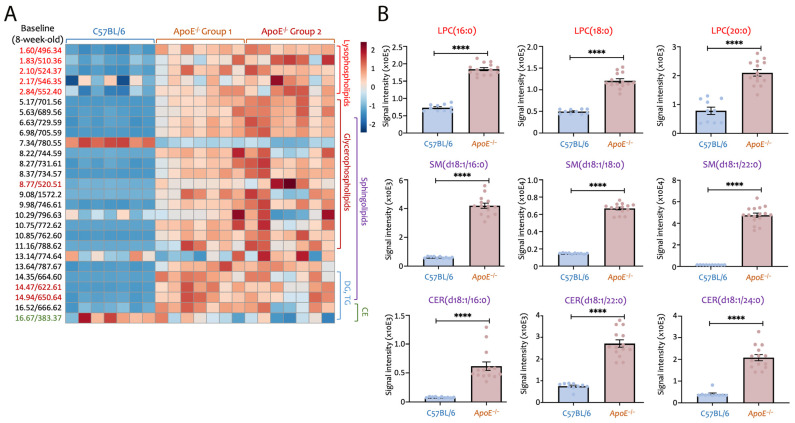
*ApoE*^−/−^ mice had altered plasma lipid patterns at 8 weeks of age, with higher levels of lysophosphatidylcholine (LPC), sphingomyelin (SM), and ceramide (CER). Data was presented in (**A**) a heatmap and (**B**) bar plots with dots. Similar to Figure 2, but the bar plots here combined data from the two *apoE*^−/−^ groups and was in signal intensity. The LPC(16:0) and LPC(18:0) were the most abundant LPC species, whereas the SM(d18:1/22:0) was the most dominant sphingolipid species in the 8-week-old mice. The data are presented in signal intensity. **** *p* < 0.0001. Abbreviations: *apoE*^−/−^: *apolipoprotein E* knockout; LPC: lysophosphatidylcholine; SM: sphingomyelin; CER: ceramide.

At the beginning of this study, we analyzed the plasma untargeted lipid profiling by liquid chromatography/mass spectrometry (LC/MS^E^) and selected signals that had an intensity greater than 1000 (higher abundance) as specific lipid markers. A total of 27 markers were chosen covering all the different lipid classes (Figure 2A). Upon analysis, the levels of LPC(16:0), LPC(18:0), LPC(20:0), SM(d18:1/16:0), SM(d18:1/18:0), SM(d18:1/22:0), CER(d18:1/16:0), CER(d18:1/22:0), and CER(d18:1/24:0) were elevated in the plasma of *apoE*^−/−^ mice (Figure 2B). Both groups of *apoE*^−/−^ mice showed no difference at the baseline. The LPC(16:0) and LPC(18:0) were the most abundant LPC species, whereas the SM(d18:1/22:0) was the most dominant sphingolipid species in the 8-week-old mice (Figure A1).

### 2.5. Profiling Dominant Lipid Species after 8 Weeks of Respective Diet Treatment (at 16th Week)

**Figure 3:**
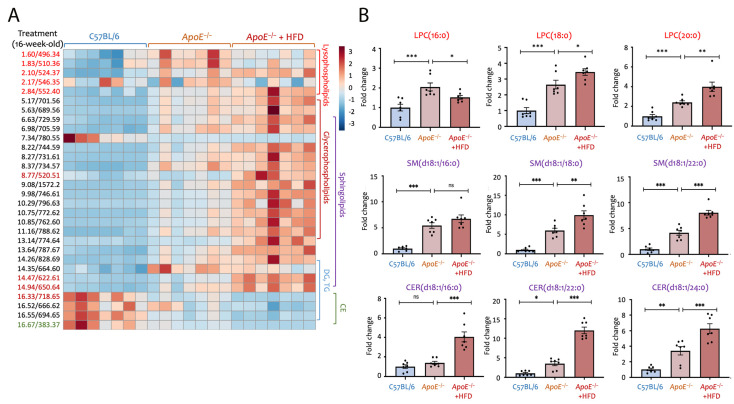
Elevated levels of sphingomyelin and ceramide in high-fat diet-fed *apoE*^−/−^ mice. The analysis of untargeted lipidomic data was carried on as described earlier. (**A**) A total of 30 markers were picked from each category and displayed in a heatmap. Of the 30 markers, 27 were the same as those identified at the baseline. (**B**) LPC(16:0), LPC(18:0), LPC(20:0), SM(d18:1/16:0), SM(d18:1/18:0), SM(d18:1/22:0), CER(d18:1/16:0), CER(d18:1/22:0), and CER(d18:1/24:0) were the most abundant lipid species in each category. Levels of each lipid species were compared among groups (N = 7 each). The information is provided in the fold change format. *** *p* < 0.001, ** *p* < 0.01, * *p* < 0.05, ns: not significant between groups. Abbreviations: *apoE*^−/−^: *apolipoprotein E* knockout; HFD: high-fat diet; NCD: normal-chow diet; LPC: lysophosphatidylcholine; SM: sphingomyelin; CER: ceramide.

**Figure A2:**
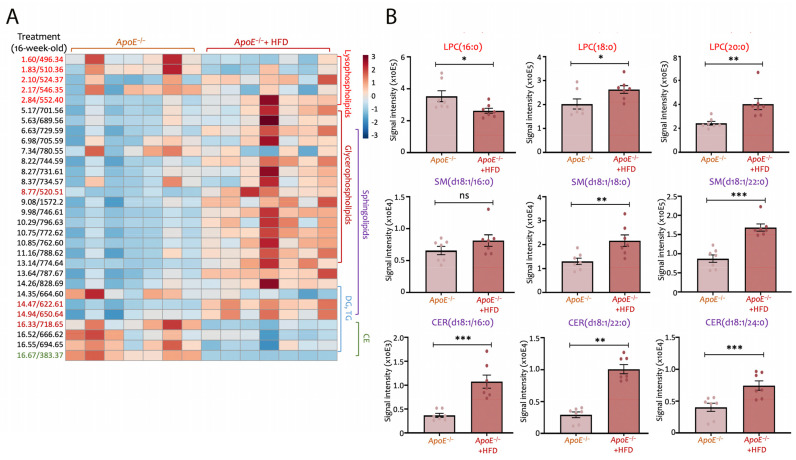
High-fat diet-fed *apoE*^−/−^ mice show enhanced levels of sphingomyelin and ceramide. Data was presented in (**A**) a heatmap and (**B**) bar plots with dots in signal intensity. Similar to Figure 3, the 16-week-old mice showed that LPC(16:0), LPC(18:0) and SM(d18:1/22:0) remain the most dominant LPC and sphingolipid species in the plasma, respectively. *** *p* < 0.001, ** *p* < 0.01, * *p* < 0.05, ns: not significant between groups. Abbreviations: *apoE*^−/−^: *apolipoprotein E* knockout; LPC: lysophosphatidylcholine; SM: sphingomyelin; CER: ceramide; HFD: high-fat diet.

**Figure A3:**
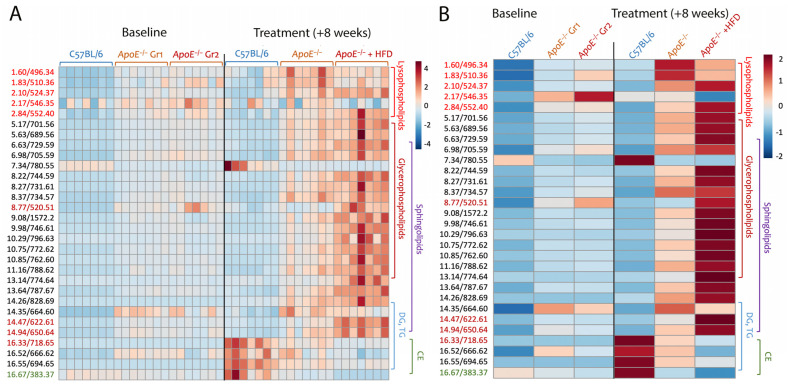
*ApoE*^−/−^ mice fed with a high-fat diet showed higher SM and CER levels. Data was displayed in heatmaps (**A**) individually and (**B**) average of n = 7. We accessed the disease model’s trajectory using all of the lipid profile data from mice plasma. Our findings showed that *apoE*^−/−^ mice had increased lysophospholipid levels regardless of whether they were fed NCD or HFD. LPC levels in *apoE*^−/−^ mice were two to four times greater than in C57BL/6 mice, whether they were 8 or 16 weeks old. The results demonstrated that *apoE*^−/−^ mice exhibited higher lysophospholipid levels regardless of whether they were administered NCD or HFD. LPC levels were two to four times higher in *apoE*^−/−^ mice compared to C57BL/6 mice, regardless of whether they were 8 or 16 weeks old. Sphingolipids such as SM and CER were determined to be significantly higher in *apoE*^−/−^ mice when administered HFD. At the baseline or 16-week-old, SM and CER were elevated three- to five-fold in *apoE*^−/−^ mice that compared to C57BL/6. After HFD treatment, the difference could be more than ten times elevated in comparison to C57BL/6. Abbreviations: *apoE*^−/−^: *apolipoprotein E* knockout; HFD: high-fat diet; NCD: normal-chow diet; LPC: lysophosphatidylcholine; SM: sphingomyelin; CER: ceramide.

Following 8 weeks of applying the respective diets, a total of 30 markers encompassing all lipid classes were chosen (Figure 3A). LPC(18:0), LPC(20:0), SM(d18:1/18:0), SM(d18:1/22:0), CER(d18:1/16:0), CER(d18:1/22:0), and CER(d18:1/24:0) levels were significantly higher in the plasma of *apoE*^−/−^ mice given HFD compared to *apoE*^−/−^ mice with NCD (Figure 3B). However, LPC(16:0) levels were significantly lower in the HFD group of *apoE*^−/−^ mice compared to the NCD group, whereas there was no difference in SM(d18:1/16:0) levels between the HFD and NCD groups (Figure 3B). It is worth mentioning that LPC(16:0), LPC(18:0), and SM(d18:1/22:0) remain the most dominant LPC and sphingolipid species in the plasma of 16-week-old mice (Figure A2 and Figure A3).

### 2.6. Effect of High-Fat Diet on Cholesteryl Ester Levels in apoE^−/−^ Mice

**Figure 4:**
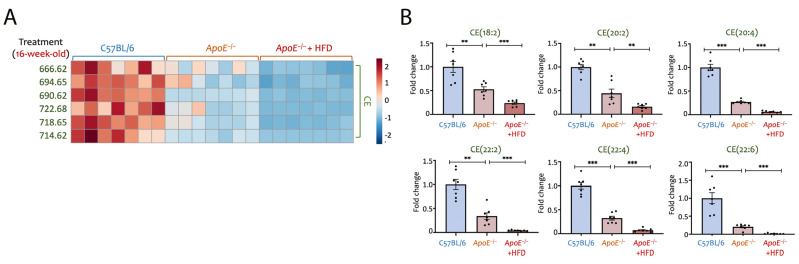
Analysis of cholesteryl ester levels in high-fat diet-fed *apoE*^−/−^ mice. (**A**) By the exact mass and retention time, CEs were analyzed similarly to other lipid parameters. CE(18:2), CE(20:2), CE(20:4), CE(22:2), CE(22:4), and CE(22:6) were the most abundant CE species in mice. CE levels were compared among three groups. Data were presented in a heatmap. (**B**) CE(18:2), CE(20:2), CE(20:4), CE(22:2), CE(22:4), and CE(22:6) were all reduced significantly in *apoE*^−/−^ mice given NCD or HFD. Notably, the CE levels were lowest in *apoE*^−/−^ mice on a high-fat diet. The data are presented in the fold change. *** *p* < 0.001, ** *p* < 0.01. Abbreviations: *apoE*^−/−^: *apolipoprotein E* knockout; HFD: high-fat diet; NCD: normal chow diet; CE: cholesteryl ester.

**Figure A4:**
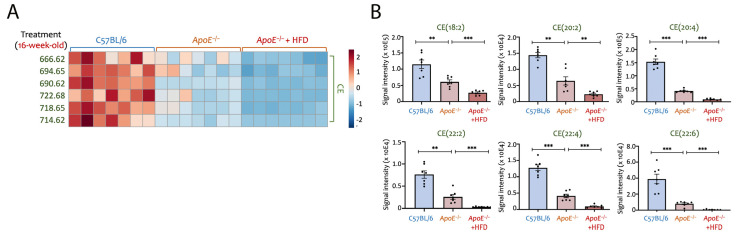
High-fat diet-fed *apoE*^−/−^ mice showed decreased cholesteryl ester levels. Data was presented in (**A**) a heatmap and (**B**) bar plots with dots in signal intensity. Similar to Figure 4 but presented with signal intensity, the 16-week-old mice showed that CE(18:2) and CE(20:4) were the most dominant CE species in the plasma of *apoE*^−/−^ mice. All CE species were down-regulated in NCD- or HFD-fed *apoE*^−/−^ mice. *** *p* < 0.001, ** *p* < 0.01. Abbreviations: *apoE*^−/−^: *apolipoprotein E* knockout; HFD: high-fat diet; NCD: normal-chow diet; CE: cholesteryl ester.

Despite the fact that levels of total cholesterol were highly elevated in Group II and Group III (Table 2), we observed down-regulated levels of cholesteryl esters (CEs) in NCD- or in HFD-fed *apoE*^−/−^ mice (Figure 4A). Progenesis QI analysis revealed that cholesteryl esters species included CE(18:2), CE(20:2), CE(20:4), CE(22:2), CE(22:4), and CE(22:6). In other words, the CE levels were highest in Group I mice and were lowest in Group III mice (Figure 4). The signal intensity of each CE species is depicted in Figure A4.

## 3. Discussion

*ApoE*^−/−^ mouse fed with HFD is a widely accepted animal model. Moreover, the pathophysiological events occurring in this model are similar to those in humans [38]. As atherosclerosis is noted for altered lipid metabolism, elucidating the exact lipid profile pattern in this model is of pivotal importance while studying the progression of the disease and device innovative treatment strategies.

In this study, the expected deletion of the *apoE* gene altered the lipid homeostasis as revealed by the altered lipid parameters even at the baseline level (Table 1). Upon feeding HFD, almost all the parameters relevant to atherosclerosis were elevated (Table 2). Our findings are comparable with previous studies that reported severe hypercholesterolemia and atherosclerosis in *apoE*-deficient mice, which worsened when fed a high-fat Western-type diet [39,40]. ApoE plays a critical role in plasma lipid homeostasis, and lipoprotein clearance [41]. In addition, apoE collaborates with ATP binding cassette subfamily A member 1 (ABCA1) in HDL synthesis and reverses cholesterol transport from peripheral tissues to the liver [42].

Among the lipid parameters studied, TG was 1.8 folds higher in the plasma of *apoE*^−/−^ mice at baseline and 2.4-fold higher after 8 weeks HFD feeding (Table 1 and Table 2). TG is synthesized in the liver through the glycerol-3-phosphate (G3P) pathway. G3P acyltransferase (GPAT) may esterify acyl-CoA molecules with G3P to produce lysophosphatidic acid (LPA), which controls the rate-limiting step of TG synthesis [43]. The enhancement of hepatic GPAT activity and expression is based on insulin through sterol regulatory element-binding protein-1 (SREBP-1) [44]. TG accumulation has been linked to higher de novo lipogenesis or fatty acid absorption in the liver. Furthermore, apoE deficiency inhibits VLDL metabolism and causes TG buildup in the plasma [43].

The noteworthy observation of this study was that LPC, SM, and CER levels were higher in the plasma of *apoE*^−/−^ mice. LPC levels in *apoE*^−/−^ mice were two to four times greater in wild-type mice of the same age (Figure 2 and Figure 3). Lp-PLA2 (lipoprotein-associated phospholipase A2) hydrolyzes phosphatidylcholine and generates LPC in the circulation, which triggers vascular inflammation [25]. Several reports demonstrated that Lp-PLA2 correlates with hypercholesterolemia and hyperlipidemia [45,46]. In addition, both LPC and Lp-PLA2 are linked to CVD [47]. LPC impairs mitochondrial integrity and enhances cytochrome C release in hepatocytes [48]. Subsequently, mitochondrial dysfunction induces triglyceride accumulation in the liver [49]. On the other hand, oxidative stress or HFD increases hypoxia-inducible factor-1α (HIF-1α) and leads to LPC overproduction in the liver [25]. Reversely, a high level of LPC enhances HIF-1α expression and triggers hypoxia-response elements, forming a positive autoregulatory loop. The aforementioned process is linked to LDL(−) formation and contributes to inflammation (paper under review).

Both at baseline and after diet therapy, *apoE*^−/−^ mice (Group II and Group III) had higher levels of LPC than wild-type controls (Group I). These results were not surprising, as shown previously by Liu et al. where *apoE*^−/−^ fed on HFD cleared [^3^H] PAF more slowly than their wild-type control, due to competition by truncated oxidized phospholipids [50]. However, it was unexpected that the LPC(16:0) was not the same as in other LPC species elevated in the plasma of HFD-fed *apoE*^−/−^ mice in comparison to the NCD group. The explanation for this point needs further investigation.

Levels of sphingolipids such as SM and CER increased significantly in HFD-fed *apoE*^−/−^ mice (Figure 3). Endoplasmic reticulum (ER) is the organelle responsible for sphingolipid biogenesis and sterol production, notably the predominant enzyme ceramide synthase (CerS), which controls the de novo ceramide pathway [51]. When ER homeostasis is disturbed due to increased lipid availability and ceramide accumulation, ER stress contributes to obesity and insulin resistance [52]. In addition, CER triggers immune cell activation and enhances vascular endothelial cell apoptosis [26,51]. CER can be metabolized primarily by lysosomal acid ceramidase into fatty acid and sphingosine, a precursor of the antiapoptotic molecule sphingosine-1-phosphate [53]. Furthermore, CER can be converted to SM in the Golgi apparatus or plasma membranes by sphingomyelin synthase [51].

Elevated blood cholesterol levels have long been established as risk factors for CVDs [54]. Cholesterol in plasma exists in two forms: free cholesterol and cholesteryl esters (CEs). Esterification of free cholesterol to CE occurs in the endoplasmic reticulum of both intestinal and liver cells and is mediated by an acyl-coenzyme A:cholesterol acyltransferase (ACAT). Based on our results, total cholesterol level was 3.2 times higher in NCD-fed *apoE*^−/−^ mice and 8 times higher in HFD-fed *apoE*^−/−^ mice compared to C57BL/6 mice (Table 2). However, regardless of NCD or HFD group, all species of CE were decreased in *apoE*^−/−^ mice. Notably, CEs were much lower in *apoE*^−/−^ mice fed with a high-fat diet (Figure 4). These findings have never previously been reported. Further study is needed to determine the mechanism of free cholesterol conversion to CE in *apoE*^−/−^ animals given HFD. Nevertheless, in humans, plasma LPC levels increased while the ratio of CE to free cholesterol declined in the blood of cardiovascular patients. The authors concluded that the patients’ cholesterol is inefficiently converted to CEs [54].

The *apoE*^−/−^ mouse model has been frequently used in drug research, particularly in preclinical trials of cholesterol-lowering medications such as statins and PCSK9 inhibitors [55]. When combined with HFD feeding, TG and LDL-C levels can be exceedingly high. Until now, the underlying mechanism of atherogenesis mainly centered on oxidative modification of LDL. We and our collaborators previously demonstrated the presence of LDL(−) in the circulation of hyperlipidemic individuals and animal models, while others used in vitro modified oxidized LDL [22,56]. In vitro oxidized LDL has been shown to activate the endothelial, smooth muscle, and immune cells. Cholesterol-lowering medications cannot eliminate modified atherogenic lipids from circulation, and ASCVD continues to be the leading cause of death worldwide [57].

In conclusion, the levels of LPC and CER in HFD-fed *apoE*^−/−^ mice are elevated when compared to those of their wild-type counterparts. Thus, LPC and CER may serve as new diagnostic markers for the progression of atherosclerosis, and lowering these may offer a new therapeutic approach against ASCVD. The *apoE*^−/−^ model suggests that it may be effective for developing LPC- and CER-lowering ASCVD therapeutics in addition to statin and PCSK9 therapy.

## 4. Materials and Methods

### 4.1. Materials

1-palmitoyl-2-hydroxy-*sn*-glycero-3-phosphocholine (C16:0 LPC), 1-stearoyl-2-hydroxy-*sn*-glycero-3-phosphocholine (C18:0 LPC), N-palmitoyl-D-erythro-sphingosylphosphorylcholine (d18:1/16:0 SM), N-stearoyl-D-erythro-sphingosylphosphorylcholine (d18:1/18:0 SM), N-palmitoyl-D-erythro-sphingosine (d18:1/16:0 ceramide), N-lignoceroyl-D-erythro-sphingosine (d18:1/24:0 ceramide) and ceramide/sphingolipid internal standard mixture I (contains C17 base sphingosine, C17 base sphinganine, C17 base sphingosine-1-P, C17 base sphinganine-1-P, lactosyl(ß) C12 ceramide, 12:0 sphingomyelin, glucosyl(ß) C12 ceramide, 12:0 ceramide, 12:0 ceramide-1-P, 25:0 ceramide) were purchased from Avanti Polar Lipids (Alabaster, AL, USA). Standard stock solutions were dissolved in chloroform/methanol 1:1 (*v/v*) at a concentration of 1 mM and stored at −20 °C. Lipid standard mixtures were prepared freshly every day in lipid buffer (2:1:1; Isopropanol, LC/MS Grade Merck Millipore, MA, USA; Acetonitrile; H_2_O, LC/MS reagent, 9831-2, J.T.Baker, Phillipsburg, NY, USA) at a concentration of 25 μM and used immediately.

### 4.2. Animals

The Institutional Animal Care and Use Committee (IACUC) of Kaohsiung Medical University (KMU) approved these experiments (IACUC-109273). All tests were conducted in compliance with the institution’s regulations, using humane endpoints and adhering to ethical principles. Six-week-old male C57BL/6 (wild type) and *apoE*^−/−^ mice (C57BL/6-Apoe^em1Narl/Narl^) were purchased from National Applied Research Laboratories (NARL) Taiwan. Animals were kept in pathogen-free environments. Animals were acclimated until they were 8 weeks old. Following this, animals were divided into three groups Group I—C57BL/6 wild-type (n = 7) fed on the normal-chow diet (NCD), Group II— *apoE*^−/−^ (n = 7) fed on NCD (Research Diets Inc., New Brunswick, NJ; D12450B, USA) and Group III— *apoE*^−/−^ (n = 7) fed with HFD. The HFD was procured from Research Diets Inc. (D12492; New Brunswick, NJ USA) and comprised 60 kcal% fat, 20 kcal% protein, and 20 kcal% carbohydrates per 100 g of pellet. Blood was drawn into anti-coagulated (K2-EDTA) collection tubes from the tail vein at baseline (8 weeks old) and after 8 weeks (16 weeks old).

### 4.3. Biochemical Profile

Whole blood was gently centrifuged for 15 min at 800× *g* at 4 °C with soft braking. Plasma was collected after centrifugation for biochemical analysis including aspartate aminotransferase (AST), alanine aminotransferase (ALT), blood urea nitrogen (BUN), creatinine (Cr), glucose, total cholesterol (TC), triglyceride (TG), high-density lipoprotein-cholesterol (HDL-C), and low-density lipoprotein-cholesterol (LDL-C). The NARL Taiwan performed these tests using a Hitachi 7080 biochemical analyzer.

### 4.4. Lipid Extraction

Plasma lipids were extracted using the Folch technique [58,59]. In summary, 20 μL aliquots of plasma were mixed with 780 μL MS grade water (PURELAB Option-Q, UK), followed by 2 mL methanol (A456-4, LC/MS Grade, Thermo Fisher Scientific, Waltham, MA, USA) and 1 mL chloroform (9831-2, LC/MS reagent, J.T.Baker, Phillipsburg, NY, USA) in the ratio of 2:1:0.8 (methanol:chloroform:sample). The mixture was vortexed for 15 s. Then, another 1 mL of MS-grade water was added along with 1 mL of chloroform, and then vortexed. The sample tubes were centrifuged at 800× *g*, 4 °C for 10 min. The upper aqueous solvent was discarded, and the lower organic phase was collected into a new glass vial, which was then evaporated with a nitrogen gas evaporator (Eyela MG-2200; Japan). It was then solubilized using sample buffer (2:1:1; Isopropanol, LC/MS Grade Merck Millipore, MA, USA; Acetonitrile; H_2_O, LC/MS reagent, 9831-2, J.T.Baker, Phillipsburg, NY, USA) and stored at −80 °C until LC/MS^E^ analysis.

### 4.5. LC/MS^E^ Analysis for Lipid Composition

Total lipids were extracted and quantified using a liquid chromatography data-independent parallel-fragmentation mass spectrometry (LC/MS^E^) system equipped with a charged surface hybrid (CSH™) C18 column [28,60]. Briefly, lipids were extracted and separated using an ACQUITY^®^ UPLC (Waters Corporation, Milford, MA, USA) equipped with CSH™ 1.7 um, 2.1 mm × 10 cm C18 column under gradient conditions at a flow rate of 0.1 mL/min for 20 min at 55 °C. The mobile phase A was composed of 10 mM ammonium formate (Merck Millipore, Burlington, MA, USA) and 0.1% (*v/v*) formic acid (Merck Millipore, MA, USA) in acetonitrile (ACN) (Merck Millipore, MA, USA) /H_2_O in the ratio 60:40. Mobile phase B contained 10 mM ammonium formate and 0.1% (*v/v*) formic acid in isopropanol (IPA) (Merck Millipore, MA, USA)/ACN in the ratio 90:10 for molecule protonation. Mass spectrometry was carried out using a Xevo G2 QTOF (quadrupole time of flight mass spectrometry; Waters Corporation, Milford, MA, USA) equipped with an electrospray ionization interface (ESI) and operated in the data-independent collection mode (MS^E^). Parallel ion fragmentation had been programmed to switch between low (4 eV) and high (35–55 eV) energies in the collision cell, and signals were collected from 250 to 1600 *m/z* utilizing leucine enkephalin (556.2771 Da) as the separate data channel lock mass calibrant. Data were processed by Progenesis QI^®^ (Waters Corporation; Milford, MA, USA). Analysis modules were enhanced by updating chemical identifiers from LipidBlast [61], PubChem [62] and ChEBI [63]. We expanded the database by including additional lipids from LIPID MAPS [64]. The data were processed and visualized using MetaboAnalyst 5.0 [65].

### 4.6. Statistical Analyses

For discrete responses, all data are provided as relative frequencies, and for continuous responses they are provided as the mean and standard error of the mean (SEM). GraphPad Prism Software version 9.2.0 was used for statistical analysis and statistical graphics. The differences between groups were evaluated using the two-tailed student *t*-test, the Chi-square goodness of fit test, the Mann–Whitney U test, the one-way analysis of variance with Bonferroni’s post hoc test, the Kruskal–Wallis test of variance with Dunn’s post hoc test, and Pearson’s correlation as indicated in the respective legends. *p* < 0.05 is considered statistically significant. The following statistical significance levels are shown for C57BL/6 versus *apoE*^−/−^ or *apoE*^−/−^ with NCD versus HFD: * *p* < 0.05, ** *p* < 0.01, *** *p* < 0.001.

## 5. Conclusions

Both bioactive lipids LPC and CER were significantly elevated in *apoE*^−/−^ mice, while the latter was substantially higher following HFD feeding. We conclude that HFD-fed *apoE*^−/−^ mice are a suitable model for developing LPC- and CER-lowering ASCVD therapeutics. These bioactive lipids could play crucial roles in the etiology of ASCVD. The noteworthy findings of this study provide new insights into the treatment of ASCVD.

## Figures and Tables

**Table 1:** Biochemical parameters in all the three groups of mice at their baseline.

Baseline (8 Weeks Old)					
	Wild-Type Group I (A)	*ApoE*^−/−^ Group II (B)	*ApoE*^−/−^ Group III (C)	*p* Value (A vs. B)	*p* Value (B vs. C)
Weight (g)	20.66 ± 0.47	22.99 ± 0.65	23.19 ± 0.83	*	NS
AST (U/L)	38.89 ± 1.27	45.39 ± 3.74	53.13 ± 2.65	NS	NS
ALT (U/L)	18.81 ± 0.32	21.01 ± 2.21	26.54 ± 2.33	NS	NS
Glucose (mg/dL)	154.47 ± 5.92	185.93 ± 9.11	184.29 ± 7.29	**	NS
BUN (mg/dL)	19.77 ± 0.86	25.73 ± 1.79	25.64 ± 1.21	*	NS
Cr (mg/dL)	0.06 ± 0.00	0.06 ± 0.00	0.06 ± 0.00	NS	NS
TC (mg/dL)	145.46 ± 10.87	431.49 ± 37.47	447.26 ± 36.82	***	NS
TG (mg/dL)	88.04 ± 2.79	158.42 ± 19.26	158.75 ± 32.50	***	NS
HDL-C (mg/dL)	45.74 ± 1.13	54.11 ± 3.00	54.69 ± 2.50	*	NS
LDL-C (mg/dL)	194.04 ± 4.50	282.01 ± 25.57	297.50 ± 24.26	**	NS
LDL/HDL	4.26 ± 0.15	5.23 ± 0.45	5.44 ± 0.39	NS	NS

* All plasma biochemical parameters were detected by Hitachi 7080. Values are provided as means ± SEM. N = 7 in each group. *** *p* < 0.001, ** *p* < 0.01, * *p* < 0.05. A vs. B represents Group I versus Group II at baseline; B versus C represents Group II versus Group III at baseline. Abbreviations: HFD: high-fat diet; NCD: normal-chow diet; Weight: body weight; AST: aspartate aminotransferase; ALT: alanine aminotransferase; BUN: blood urea nitrogen; Cr: Creatinine; TC: total cholesterol; TG: triglyceride; HDL-C: high-density lipoprotein cholesterol; LDL-C: low-density lipoprotein cholesterol; NS: not significant.

**Table 2:** Biochemical parameters after 8 weeks of respective treatment in all the three groups of mice.

Treatments (+8 Weeks)					
	Wild-Type Group I NCD (A)	*ApoE*^−/−^ Group II NCD (B)	*ApoE*^−/−^ Group III HFD (C)	*p* Value (A vs. B)	*p* Value (B vs. C)
Weight (g)	25.56 ± 0.26	26.22 ± 0.72	32.04 ± 1.01	NS	***
AST (U/L)	40.10 ± 1.10	52.60 ± 14.40	128.50 ± 41.40	NS	*
ALT (U/L)	21.00 ± 0.40	23.20 ± 2.50	37.90 ± 2.20	NS	***
Glucose (mg/dL)	197.90 ± 5.10	223.60 ± 9.60	234.80 ± 7.50	*	NS
BUN (mg/dL)	21.20 ± 1.10	29.40 ± 2.30	20.10 ± 0.40	*	*
Cr (mg/dL)	0.06 ± 0.00	0.07 ± 0.01	0.13 ± 0.02	NS	**
TC (mg/dL)	154.80 ± 15.60	495.00 ± 29.00	1243.40 ± 48.00	***	***
TG (mg/dL)	100.00 ± 4.30	144.30 ± 13.10	230.70 ± 25.40	**	*
HDL-C (mg/dL)	45.30 ± 2.20	46.50 ± 2.50	70.50 ± 3.10	NS	***
LDL-C (mg/dL)	221.00 ± 14.90	325.30 ± 21.30	1269.10 ± 50.30	**	***
LDL/HDL	4.92 ± 0.36	7.06 ± 0.48	18.22 ± 1.07	**	***

* All plasma biochemical parameters were detected by Hitachi 7080. Values are provided as means ± SEM. N = 7 in each group. *** *p* < 0.001, ** *p* < 0.01, * *p* < 0.05. A vs. B represents Group I versus Group II after 8-week treatment with normal-chow diet; B versus C represents Group II (treated with normal-chow diet for 8 weeks) versus Group III (treated with high-fat diet for 8 weeks). Abbreviations: HFD: high-fat diet; NCD: normal-chow diet; Weight: body weight; AST: aspartate aminotransferase; ALT: alanine aminotransferase; BUN: blood urea nitrogen; Cr: Creatinine; TC: total cholesterol; TG: triglyceride; HDL-C: high-density lipoprotein cholesterol; LDL-C: low-density lipoprotein cholesterol; NS: not significant.

## Data Availability

The data that support the findings of this study are available in the methods and/or Appendix A of this article.

## Abbreviations

ABCA1	ATP binding cassette subfamily A member 1
ACAT	acyl-coenzyme A: cholesterol acyltransferase
ACN	acetonitrile
Acyl-CoA	acyl-coenzyme A
ALT	alanine aminotransferase
ApoE	apolipoprotein E
*apoE* ^−/−^	apolipoprotein E-deficient
ANGPTL3	angiopoietin-like protein 3
ASCVD	atherosclerotic cardiovascular disease
AST	aspartate aminotransferase
ATP	adenosine triphosphate
BUN	blood urea nitrogen
CerS	ceramide synthase
CE	cholesteryl ester
CVD	cardiovascular disease
Cr	creatinine
CSH	charged surface hybrid
DG	diacylglycerol
EDTA	ethylenediaminetetraacetic acid
ER	endoplasmic reticulum
ESI	electrospray ionization interface
GOT	glutamic oxaloacetic transaminase
G3P	glycerol-3-phosphate
GLP-1	glucagon-like peptide 1
GPAT	glycerol-3-phosphate acyltransferase
GPT	glutamic pyruvic transaminase
HDL-C	high-density lipoprotein cholesterol
HFD	high-fat diet
HIF-1α	hypoxia-inducible factor-1α
IACUC	Institutional Animal Care and Use Committee
IPA	isopropanol
K2-EDTA	two chelated potassium ions EDTA
L5-LDL	the most electronegative subfraction of low-density lipoprotein
LDL(−)	electronegative low-density lipoprotein
LDL-C	low-density lipoprotein-cholesterol
LDLR	low-density lipoprotein receptor
LC/MS	liquid chromatography/mass spectrometry
LC/MS^E^	liquid chromatography/ data-independent collection mode mass spectrometry
LOX-1	lectin-like oxidized low-density lipoprotein receptor 1
LPA	lysophosphatidic acid
LPC	lysophosphatidylcholine
Lp-PLA2	lipoprotein-associated phospholipase A2
MS	mass spectrometry
MS^E^	data-independent collection mode mass spectrometry
NADPH	nicotinamide adenine dinucleotide phosphate (reduced)
NARL	National Applied Research Laboratories
NCD	normal-chow diet
Nox2	NADPH oxidase 2
NOXA1	NADPH oxidase activator 1
oxLDL	oxidized low-density lipoprotein
PCSK9	Proprotein Convertase Subtilisin Kexin Type 9
ROS	reactive oxygen species
QTOF	quadrupole time of flight mass spectrometry
SEM	standard error of the mean
SM	Sphingomyelin
SREBP-1	sterol regulatory element-binding protein-1
SGLT2	sodium–glucose cotransporter 2
TC	total cholesterol
TG	triglyceride
UPLC	ultra-performance liquid chromatography

## Appendix A

**Table A1:** Lipids Classification.

Classification	Major Adducts	RT Range (min)
*Lysophospholipids*		
LPC	[M + H]^+^, [M + Na]^+^	1.33–4.77
*Glycerophospholipids*		
PC	[M + H]^+^	4.39–11.16
*Sphingolipids*		
SM	[M + H]^+^	5.17–14.32
CER	[M + H]^+^, [M + H—H_2_O]^+^	8.77–14.94
*Sterol lipids*		
CE	[M + NH_4_]^+^, [M + Na]^+^	16.18–17.17
*Glycerolipids*		
DG	[M + H—H_2_O]^+^, [M + NH_4_]^+^	13.35–15.69
TG	[M + H]^+^	14.01–16.43

The plasma lipids classification for C57BL/6 and *apoE*^−/−^ mice. Abbreviations: RT: retention time; LPC: lysophosphatidylcholine; PC: phosphatidylcholine; SM: sphingomyelin; CER: ceramide; CE: cholesteryl ester; DG: diglyceride; TG: triglyceride.
